# RNA signaling in skeletal muscle: the central role of microRNAs and exosomal microRNAs

**DOI:** 10.3389/fcell.2025.1639123

**Published:** 2025-08-04

**Authors:** Shunshun Liu, Huan Dong

**Affiliations:** School of Physical Education, Zaozhuang University, Zaozhuang, China

**Keywords:** microRNA, exosome, skeletal muscle, epigentic, aging

## Abstract

Skeletal muscle development and adaptation are governed by complex regulatory networks that coordinate gene expression, signaling pathways, and intercellular communication. Among the emerging key regulators are microRNAs (miRNAs) and exosomal microRNAs, which function as critical modulators of skeletal muscle growth, differentiation, regeneration, and metabolic adaptation. The review explores the acknowledged contributions of miRNAs, both intracellular and those encapsulated within exosomes, to the regulation of skeletal muscle physiology. We highlight their involvement in major molecular pathways, including PI3K/Akt/mTOR, TGF-β/Smad, Wnt/β-catenin, and AMPK signaling, and their impact on processes such as myogenesis, hypertrophy, atrophy, and mitochondrial function. Emphasis is placed on the critical role of exosomal miRNAs in orchestrating signaling pathways that enable communication among cells in the muscle milieu and with peripheral tissues. Ultimately, the review addresses the clinical relevance of miRNAs, including those derived from exosomes, emphasizing their prospective roles as diagnostic tools and intervention points in muscle-related conditions. In sum, the review elucidates the broad landscape of RNA-related regulatory processes in skeletal muscle and projects forward-looking strategies for translational exploration in this rapidly developing scientific domain.

## Introduction

Making up close to 40% of human body mass, skeletal muscle is integral to locomotor activity, postural control, and the orchestration of metabolic functions at the systemic level (Ju et al., 2015). Skeletal muscle, while primarily mechanical in function, also displays exceptional adaptive capacity, permitting structural, functional, and dimensional remodeling when exposed to influences such as physical training, dietary modulation, and pathological conditions (Güller and Russell, 2010; Matsakas and Patel, 2009). The modulation of gene expression driving this plasticity is managed by elaborate molecular systems, among which microRNAs (miRNAs), a class of non-coding RNAs, have been identified as crucial regulators (Ju et al., 2015; Velez, 2023; Prabhakaran et al., 2024).

MicroRNAs, characterized by their endogenous origin and short length of around 22 nucleotides, modulate gene expression by targeting mRNAs and influencing their post-transcriptional fate (Kirby et al., 2015). By targeting the 3′untranslated region (UTR) of messenger RNAs (mRNAs), microRNAs facilitate post-transcriptional gene silencing through mechanisms that either hinder translation or accelerate mRNA degradation (Kirby et al., 2015; Mayr, 2017). This mode of regulation permits miRNAs to delicately control the expression levels of many genes, influencing extensive biological activities across both normal physiology and pathological conditions (Kirby et al., 2015; Schratt, 2009).

Recent advances have underscored not only the role of intracellular miRNAs but also the importance of exosomal microRNAs, which represent a unique subclass of miRNAs encapsulated within extracellular vesicles. These vesicles, primarily exosomes, facilitate intercellular communication by delivering miRNA cargo from donor to recipient cells, influencing gene expression at a distance (Valadi et al., 2007; Fatima and Nawaz, 2017). In the context of skeletal muscle, exosomal miRNAs are secreted both constitutively and in response to stimuli such as exercise, injury, or disease, thereby participating in tissue remodeling, inflammation, regeneration, and systemic signaling (Wang et al., 2025; Magliulo et al., 2022; Fleshner and Crane, 2017). By incorporating both intracellular and exosomal pathways, miRNAs contribute to a complex, multilayered network of regulatory control that orchestrates skeletal muscle development, adaptation, and pathology.

Introducing greater complexity to gene regulation is the finding of exosome-associated miRNAs. Exosomes consist of nanosized, membrane-enclosed vesicles secreted by cells into the extracellular compartmen (Aoi, 2015). These vesicles act as mediators of intercellular communication, transporting a variety of biomolecules, including miRNAs, between neighboring or distant cells and even across different organs (Aoi, 2015; Liu and Wang, 2023; Fabbri, 2018). The incorporation of miRNAs into exosomes enhances their stability outside the cell and facilitates their conveyance to recipient cells, allowing them to modulate cellular processes (Aoi, 2015; Sohel, 2016).

A detailed knowledge of the contributions of miRNAs and exosomal miRNAs to skeletal muscle growth and adaptation is pivotal for interpreting the molecular frameworks that underlie muscle physiology in both healthy and diseased states (Güller and Russell, 2010; Eisenberg et al., 2009).

Skeletal muscle disorders represent a significant and growing global health concern. Sarcopenia, characterized by the progressive loss of muscle mass and function with age, affects up to 10%–20% of individuals over 60 years old and more than 50% of those over 80, posing a major threat to functional independence and quality of life (Cruz-Jentoft and Sayer, 2019; von Haehling et al., 2010; Ardeljan and Hurezeanu, 2020). Similarly, cachexia, commonly associated with chronic conditions such as cancer, heart failure, and chronic kidney disease, contributes to increased morbidity and mortality in millions of patients worldwide (Rogers et al., 2023; Ferrer et al., 2023). Collectively, these disorders impose a substantial burden on healthcare systems and underscore the urgency of advancing research on molecular mechanisms, biomarker discovery, and therapeutic interventions that could facilitate clinical translation. The study of miRNAs—particularly exosomal miRNAs—as potential regulators and biomarkers offers a promising avenue to address this unmet clinical need.

The purpose of this report is to furnish a broad and authoritative examination of contemporary knowledge in this subject area. It will delve into the mechanisms of miRNA biogenesis and function within skeletal muscle, explore the roles of key miRNAs in myogenesis and muscle adaptation to exercise, discuss the function of exosomal miRNAs in intercellular communication and the response to exercise, examine the dysregulation of these molecules in various muscle disorders, and finally, consider their potential as therapeutic targets. Skeletal muscle’s extensive presence in the body highlights its systemic significance, making the exploration of miRNAs’ regulatory influence a crucial research priority. Furthermore, the ability of miRNAs to either promote or alleviate muscle loss underscores the complexity of these regulatory networks and the necessity for a detailed understanding in the context of therapeutic development (Jung et al., 2024; Sharma et al., 2014; Zabihi and Akhoondian, 2025).

## Mechanisms behind skeletal muscle growth and development

The anabolic properties of insulin and insulin-like growth factor 1 (IGF1) are fundamental to the regulation and continuation of growth processes at both the systemic level and within skeletal muscle. The hormones insulin and IGF1, upon receptor binding, activate phosphorylation cascades that differentially modulate the activity of proteins, enzymes, and transcription factors, facilitating either their stimulation or inhibition. The pathway orchestrates the regulation of protein synthesis and degradation, cellular proliferation and viability, along with glucose uptake and the generation of cellular energy. Insulin is produced by the pancreas, in contrast to IGF1, which is primarily synthesized in the liver under the influence of growth hormone and functions as a systemic growth factor. IGF1 is also produced by tissues outside the liver, where it exerts mainly autocrine and paracrine effects. Muscle-targeted overexpression of a locally acting IGF1 isoform in murine models demonstrates that localized IGF1 expression is essential for promoting muscle growth and regenerative capacity (Musarò et al., 2001). Among the IGF1 isoforms differing in N-terminal signal peptides (Class 1 or 2) and C-terminal E-peptides (Ea or Eb), IGF-1Ea exhibits the greatest potency in enhancing muscle mass and force production in young and aged murine subjects (Ascenzi et al., 2019). Both insulin and IGF1 contribute to the activation of the mitogen-activated protein kinase/extracellular signal-regulated kinase (RAS-MAPK-ERK) pathway alongside the PI3K–AKT-mTOR pathway. Selective activation of the PI3K–AKT pathway by a Ras mutant induces hypertrophy in transfected fibers, while a Ras mutant restricted to the ERK pathway lacks this capability (Murgia et al., 2000). Constitutive activation of AKT induces significant hypertrophy in transfected muscle fibers, an effect similarly reproduced by inducible transgenic models specific to muscle tissue (Blaauw et al., 2009; Pallafacchina et al., 2002).

The kinase mTOR functions as a central hub for protein synthesis and degradation and is modulated by insulin and IGF1 signaling. Acting as a signaling nexus, this kinase combines stimuli from hormones, cytokines, nutrients, and ATP/AMP ratios and transmits them to the translation apparatus by modulating p70S6K1, which controls ribosomal protein S6, and 4E binding protein 1 (4EBP1), which suppresses the eukaryotic translation initiation factor 4E. Simultaneously, mTOR inhibits protein breakdown by blocking autophagy via ULK1. mTOR kinase engages with various proteins to form two distinct complexes: the rapamycin-sensitive TORC1 containing Raptor, and the rapamycin-insensitive TORC2 complex containing Rictor. Genetic research has established that these two complexes perform different functions. mTORC2 is involved in glucose and lipid homeostasis, contrasting with mTORC1, which regulates anabolic processes such as protein synthesis, ribosome formation, and mitochondrial biogenesis (Liu and Sabatini, 2020). While muscle-specific deletion of Rictor does not lead to an overt phenotype, mice deficient in Raptor and mTOR exhibit stunted postnatal growth characterized by reduced fast muscle fiber size, unaffected slow fibers, and a progressive muscular dystrophy phenotype (Risson et al., 2009; Bentzinger et al., 2008). Rapamycin, which specifically targets mTORC1, consistently obstructs muscle growth under anabolic circumstances (Pallafacchina et al., 2002). Recent genetic analyses suggest that mTOR may perform some roles independently of the mTORC1 complex. In models of mechanical overload, inducible deletion of Raptor in muscle inhibits hypertrophy yet does not affect the enhanced protein synthesis observed via puromycin incorporation (You et al., 2019). The onset of a growth defect following conditional mTOR deletion and expression of catalytically inactive mTOR occurred after the first week postnatally. Compared with conditional RAPTOR knockout mice, these animals exhibit substantially greater muscle atrophy (Bentzinger et al., 2008). Although mTORC1 complex activity is heavily suppressed in transgenic mice, their muscles still grow, albeit to a lesser extent than controls. Myofiber degeneration and a myopathic phenotype present in conditional mTOR knockout and catalytically inactive mTOR transgenic mice suggest mTOR’s essential function in supporting muscle cell survival. Rather than the expected autophagy hyperactivation following mTORC1 inhibition, these animals display reduced autophagic activity, which largely drives their pathological phenotype (Risson et al., 2009; Zhang et al., 2019). A myopathic phenotype characterized by slow progression was described in mice with chronic mTORC1 activation driven by TSC1 inhibition. The impaired autophagy system, resulting from mTOR hyperactivation, was a significant factor in the phenotype exhibited by TSC1 knockout mice (Castets et al., 2013). Collectively, these results demonstrate that mTORC1 is a major contributor to muscle homeostasis but does not exclusively regulate protein synthesis, with autophagy in muscle cells controlled through both mTORC1-dependent and independent pathways.

A further major signaling pathway regulating skeletal muscle growth centers on myostatin, which is part of the transforming growth factor β (TGFβ) superfamily. The TGFβ superfamily comprises a diverse group of more than 30 secreted ligands, characterized by differential selectivity for specific receptor subtypes. In muscle biology, myostatin is the most prominent superfamily member, highlighted by the severe muscle hypertrophy seen in myostatin knockout mice (McPherron et al., 1997). The interaction of Activin/Myostatin/TGFβ proteins with plasma membrane activin type IIB and IIA receptors (ActRIIB/IIA) and TGFβ receptors (TGFβRII) triggers recruitment and activation of receptor-like kinase (ALK)-4, −7, and −5 kinases, resulting in Smad2/3 phosphorylation and the assembly of a heterotrimeric complex with Smad4. Inhibition of Smad2/3 alone suffices to enhance muscle growth, supporting the notion that genes implicated in protein turnover are targets of these transcription factors (Winbanks et al., 2012; Sartori et al., 2009). The relationship between myostatin and the AKT/mTOR pathway is highlighted by findings that rapamycin or mTOR knockdown can negate the hypertrophic effects caused by blocking myostatin (Winbanks et al., 2012; Sartori et al., 2009).

The control of muscle mass involves BMP signaling, which converges on Smad4 as one of its key pathways (Traoré et al., 2019; Sartori et al., 2013). Members of the BMP/GDF family show selective binding to type II receptors—BMP type II receptor (BMPRII), ActRIIA, and ActRIIB—and facilitate the recruitment of type I receptors such as BMPRIA (ALK3), BMPRIB (ALK6), and ACVR1 (ALK2). Ligand/Type II/Type I receptor complexes enhance phosphorylation and heterotrimerisation of Smad1/5/8 with Smad4, thereby influencing the regulation of transcription. Thus, ligands from the two superfamily subgroups, in addition to Smad4, are likely to compete for access to certain type II receptors. Regulatory mechanisms of the pathway extend to regions downstream of the receptors. Smad6 and Smad7 proteins inhibit receptor-mediated signaling pathways that activate Smad1/5/8 and Smad2/3 (Winbanks et al., 2016). In the skeletal muscle of mice, the specific ablation of Smad4 did not facilitate hypertrophy but was linked to muscle atrophy and weakness (Sartori et al., 2013). The demonstration that BMP antagonist noggin overexpression counteracts the hypertrophic effects seen in myostatin knockout mice robustly supports the concept of genetic epistasis between the activin/myostatin and BMP pathways in muscle. Follistatin induces hypertrophy by concurrently blocking myostatin signaling and stimulating Smad1/5/8 activation, consistent with observed regulatory mechanisms (Sartori et al., 2013; Winbanks et al., 2013; Davey et al., 2016). Thus, decreased myostatin/activin activity, evidenced by reduced phosphorylation of Smad2/3, facilitates Smad4 binding to phosphorylated Smad1/5/8, which may play a role in preserving muscle tissue or counteracting β-adrenergic-induced atrophic processes.

Adrenergic signaling acts as a supplementary pathway modulating muscle mass through its interaction with the AKT-mTOR signaling cascade. The hypertrophic effect of β2-adrenergic agonists, for example, clenbuterol or formoterol, on muscle is associated with increased AKT phosphorylation and is completely prevented by rapamycin (Kline et al., 1985). Recent findings support that β2-adrenergic signaling partially engages insulin/IGF1 receptor signaling and does not affect the ERK1 pathway (Gonçalves et al., 2019). The anti-proteolytic properties of the β-adrenergic agonist formoterol were completely abolished by genetic and pharmacological inhibition of insulin receptor, IGF1 receptor, PI3K, and AKT, but remained unaffected by the ERK1/2 inhibitor U0126.

It has recently been documented that FGF19 fosters muscle hypertrophy and increases grip strength by stimulating ERK signaling, despite no activation of AKT (Benoit et al., 2017). This finding stands in opposition to prior research indicating that FGF21 is essential and sufficient for inducing muscle loss (Oost et al., 2019). Among FGF ligands, FGF19 (FGF15 in mice), FGF21, and FGF23 are characterized by their inability to bind heparan sulfates, instead associating with α- or β-klotho proteins that serve as FGFR co-receptors or co-ligands. Since both FGF19 and FGF21 interact with β-klotho and activate FGFR1-4, it is anticipated that they perform similar functions.

**FIGURE 1:**
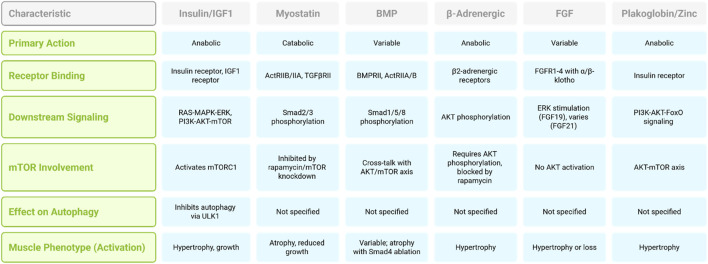
Comparison of signaling pathways regulating muscle mass.

A desmosomal protein, plakoglobin, which binds the insulin receptor and PI3K subunit p85, has recently been identified as a modulator of insulin receptor activity. The increased expression of plakoglobin enhances signaling through the PI3K–AKT-FoxO axis and drives muscle growth (Cohen et al., 2014). The relationship between zinc ions and muscle growth constitutes a significant aspect of muscle physiology. Zinc-binding metallothioneins are recognized as members of the atrogene group (see below). The inhibition of these proteins induces the release of zinc ions, which activate hypertrophic processes. The promotion of muscle growth in mice by metallothionein 2 knockdown and genetic ablation is likely mediated through the AKT-mTOR axis (Wang G. et al., 2018). Aberrant ZRT- and IRT-like protein 14 (ZIP14) expression, induced by inflammatory mediators such as TNF-α and TGF-β, results in zinc overload within muscle fibers, leading to structural damage of myosin heavy chains and subsequent muscle wasting. Notably, ZIP14 suppression in muscle has been shown to attenuate this degenerative effect in tumor-bearing experimental models (Wang G. et al., 2018; Figure 1). There are also several important signaling pathways important in muscle loss. Table 1 summarized signaling pathways involved in muscle loss.

**TABLE 1:** Summary of signaling pathways involved in muscle loss.

Signaling pathway	Key regulators	Mechanism of action	References
FoxOs-Atrogenes	FoxO1, FoxO3, FoxO4	Regulated by post-translational modifications (e.g., phosphorylation, acetylation), cofactors, and transcriptional upregulation; inhibition prevents muscle loss in various conditions	Shimizu et al. (2011), Yin et al. (2018), Raffaello et al. (2010), Brault et al. (2010), Bertaggia et al. (2012), Beharry et al. (2014), Segalés et al. (2020), O'Neill et al. (2019), Milan et al. (2015), Brocca et al. (2017)
TNF-α-IKK-IkB-NF-kB	TNF-α, IKK, IkB, NF-kB, TWEAK, Fn14	IKK activation leads to IkB degradation, NF-kB activation, MuRF1 expression; TWEAK-Fn14 axis induces NF-kB and FoxO activity	Mittal et al. (2010), Cai et al. (2004)
IL6-JAK-Stat3	IL6, JAK, STAT3	IL6 induces JAK-STAT3 signaling; Stat3 promotes muscle atrophy and upregulates atrogin-1; involved in cancer and sepsis-induced atrophy	Bonetto et al. (2012)
ATF4 and ER Stress	ATF4, PERK, eIF2α, GRP78/Bip, IRE1, XBP1	UPR activates ATF4 and XBP1, promoting atrogenes; contradictory effects observed with PERK inhibition	Ebert et al. (2012), Gallot et al. (2019), Bohnert et al. (2019)
Mitochondrial Dysfunction	OPA1, DRP1	Disruption of fusion/fission alters mitochondrial network shape, affects muscle mass regulation more than function	Favaro et al. (2019), Tezze et al. (2017)

The Wnt/β-catenin signaling pathway plays a pivotal role in embryonic myogenesis, satellite cell activation, and regeneration of adult skeletal muscle (von Maltzahn et al., 2012; Suzuki et al., 2018; Suzuki et al., 2015; Girardi and Le Grand, 2018). Activation of Wnt ligands leads to the stabilization and nuclear translocation of β-catenin, which associates with TCF/LEF transcription factors to promote the transcription of genes that drive myogenic lineage progression (Qin et al., 2024; Cadigan and Waterman, 2012; Liu et al., 2022). This pathway is essential for the proper expansion and differentiation of muscle progenitor cells. Several miRNAs have been found to modulate Wnt signaling components. For example, miR-29 targets negative regulators of Wnt signaling such as Dkk1, thereby enhancing β-catenin activity and promoting myogenic differentiation (Hsu et al., 2016; Kapinas et al., 2010). Conversely, miR-206 has been shown to suppress Wnt signaling by targeting Wnt5a, indicating that the regulatory outcome is context-dependent and tightly controlled (Yi et al., 2016; Zhou et al., 2019).

AMP-activated protein kinase (AMPK) functions as a central energy sensor that promotes catabolic processes and mitochondrial biogenesis in response to energetic stress, such as during endurance exercise (Rothschild et al., 2022; Niederberger et al., 2015; Lantier et al., 2014). AMPK activation leads to increased glucose uptake, fatty acid oxidation, and inhibition of mTORC1, thereby shifting the muscle phenotype toward oxidative metabolism (Tang et al., 2023; Marcondes‐de‐Castro et al., 2023). miRNAs also participate in regulating AMPK signaling. For instance, miR-128 negatively regulates AMPKα1, reducing the energy-sensing capacity of muscle cells, while miR-195 has been reported to target SIRT1, an upstream regulator of AMPK, thereby modulating mitochondrial function and oxidative capacity (Yuan et al., 2020; Guan et al., 2025; Kjøbsted et al., 2018; Sun and Kemper, 2023). In contrast, miR-23a promotes mitochondrial biogenesis and oxidative gene expression by suppressing PGC-1α repressors, indirectly supporting AMPK-mediated metabolic reprogramming (Krammer et al., 2022; Du et al., 2019; Wang et al., 2015).

## MicroRNA biogenesis

**FIGURE 2:**
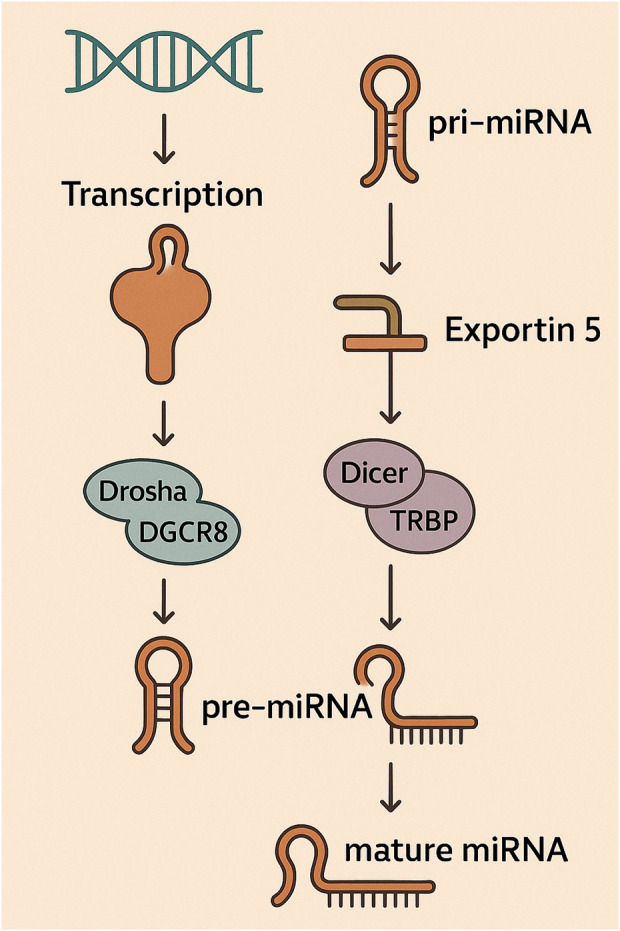
The initial step in miRNA biogenesis involves the transcription of pri-miRNA, subsequently processed by the Drosha-DGCR8 complex to form pre-miRNA. Following transport by Exportin-5 into the cytoplasm, pre-miRNA is processed by the Dicer-TRBP complex into mature miRNA, which is then incorporated into the RISC.

The synthesis of miRNAs follows a strictly regulated, sequential process that starts when RNA polymerase II commonly transcribes miRNA genes, leading to the production of extended precursor molecules referred to as primary miRNA transcripts (pri-miRNAs) (Singh et al., 2020; Catalanotto et al., 2016; Olejniczak et al., 2018). Nuclear processing of pri-miRNAs involves cleavage by a complex consisting of Drosha and DGCR8, leading to the generation of shorter precursor miRNAs (pre-miRNAs) characterized by their stem-loop structures (Singh et al., 2020; Yoshida et al., 2021; Ying, 2019). Exportin-5 plays a pivotal role in shuttling pre-miRNAs from the nucleus into the cytoplasm following their initial processing, ensuring their progression through the miRNA maturation pathway (Singh et al., 2020; Wu et al., 2018). Following cytoplasmic export, pre-miRNAs are subjected to cleavage by Dicer, an RNase III enzyme, which processes the hairpin structure to yield short, double-stranded miRNA duplexes (Singh et al., 2020; Park, 2015). The RNA-induced silencing complex (RISC), a ribonucleoprotein assembly including the Argonaute protein AGO2, selectively incorporates one strand of the duplex known as the mature miRNA (Kirby et al., 2015; Tang, 2005). The mature miRNA within RISC acts as a guide, directing the complex to target mRNAs that possess complementary sequences, primarily within their 3′UTR (Kirby et al., 2015; van den Berg et al., 2008). Binding of miRNA to target mRNA induces either translational inhibition or transcript degradation, culminating in decreased expression of the protein encoded by the mRNA (Kirby et al., 2015; Valinezhad Orang et al., 2014; Valencia-San et al., 2006). A single miRNA molecule demonstrates the capacity to influence multiple mRNA targets, typically those encoding proteins involved in coordinated cellular pathways or biological functions (Zhang and Chen, 2018; Ying et al., 2008). The intricate nature of this biogenesis pathway, involving multiple enzymatic steps and transport mechanisms, provides numerous potential points for regulation, allowing for a dynamic control of miRNA expression in response to various cellular signals and environmental cues (Singh et al., 2020; Yan et al., 2025; Figure 2).

While the majority of miRNAs are generated through the canonical Drosha-Dicer pathway, accumulating evidence has identified non-canonical routes that bypass one or more processing steps. These alternative pathways expand the regulatory potential of the miRNA landscape and have particular relevance in contexts like stress response, tissue-specific regulation, and exosomal sorting (Miyoshi et al., 2010; Abdelfattah et al., 2014; Santovito and Weber, 2022). A prominent example is miR-451, which undergoes Drosha processing to yield pre-miR-451, but bypasses Dicer cleavage (Kretov et al., 2020; Yang and Lai, 2010). Instead, it is directly cleaved by Argonaute 2 (Ago2), which also serves as its slicer and stabilizer. This pathway reflects a Dicer-independent biogenesis mechanism and appears to be conserved in erythropoiesis and skeletal muscle differentiation, where miR-451 has known regulatory roles (Kretov et al., 2020; Yang and Lai, 2010). Interestingly, miR-451 has also been observed to be enriched in exosomes, possibly reflecting distinct export preferences related to its non-canonical maturation route (Kumari et al., 2020; Guduric-Fuchs et al., 2012).

Another example is miR-133a, which, although canonically processed, is subject to additional levels of regulation through promoter-specific expression, splicing variants, and clustered transcription with miR-1 (Davis and Hata, 2009; Mitchelson and Qin, 2015). These mechanisms may influence not only its expression levels but also its incorporation into exosomes, where it is frequently detected following muscle injury or exercise.

Additional non-canonical pathways include mirtrons—miRNA precursors that arise from spliced introns and bypass Drosha cleavage. Although less studied in muscle tissue, their relevance is increasing with the discovery of new intron-derived miRNAs in muscle transcriptomic datasets (Hubé et al., 2017). Collectively, non-canonical biogenesis pathways contribute to the complexity of miRNA-mediated regulation and may partially explain the preferential inclusion of certain miRNAs into exosomes. Their study is essential for understanding both miRNA functional diversity and the selective packaging mechanisms behind intercellular communication.

Following intracellular processing, a subset of mature miRNAs is selectively packaged into exosomes—small extracellular vesicles (30–150 nm in diameter) originating from the endosomal system. This exosomal compartmentalization introduces an additional regulatory layer in miRNA biology, enabling these molecules to act not only within the originating cell but also as messengers in local and systemic communication (Han et al., 2022; Vishnoi and Rani, 2022).

Exosome biogenesis begins with the inward budding of late endosomal membranes to form multivesicular bodies (MVBs). These MVBs either fuse with lysosomes for degradation or with the plasma membrane, releasing their intraluminal vesicles as exosomes into the extracellular environment (Han et al., 2022; Vishnoi and Rani, 2022). miRNA sorting into exosomes is a non-random, actively regulated process involving several RNA-binding proteins, including hnRNPA2B1, YBX1, and SYNCRIP, which recognize specific motifs on miRNAs to mediate their selective incorporation (Corsi, 2023; Sun et al., 2023; Marocco, 2025). While the canonical miRNA biogenesis pathway culminates in the cytoplasm with RISC loading, these sorting mechanisms represent a branching fate for mature miRNAs—those not engaged in intracellular repression may instead be repurposed for extracellular delivery.

Upon secretion, exosomal miRNAs are taken up by recipient cells via endocytosis, membrane fusion, or receptor-mediated pathways (Wang et al., 2025; Liu et al., 2024). In skeletal muscle, this intercellular delivery system allows myofibers, satellite cells, fibroblasts, endothelial cells, and infiltrating immune cells to communicate dynamically during development, regeneration, and adaptation (Wang W. et al., 2022; Yue et al., 2020). Exosomes released during exercise, injury, or disease contain miRNAs that influence target cell behavior by modulating gene expression at a distance, thereby contributing to systemic crosstalk between muscle and remote tissues such as adipose, liver, and even brain (Dong et al., 2024; Luo et al., 2024).

Functionally, exosomal miRNAs have been implicated in the regulation of myogenesis, hypertrophy, atrophy, inflammation, and mitochondrial metabolism. For instance, muscle-derived exosomes carrying miR-1, miR-133a, and miR-206 have been shown to influence both local and distal responses to training or injury (Lombardo et al., 2024; Luo et al., 2024; Mytidou et al., 2021). Their presence in circulation under physiological and pathological conditions also makes them attractive candidates for non-invasive biomarkers of muscle health.

By mediating horizontal transfer of regulatory information, exosomal miRNAs expand the functional repertoire of skeletal muscle as not only a contractile organ but also a secretory tissue. This vesicle-based communication system represents a critical and emerging frontier in muscle biology, with far-reaching implications for diagnostics, therapeutics, and our understanding of tissue-level coordination.

## MicroRNAs and their exosomal forms in skeletal muscle development and adaptation

In skeletal muscle tissue, miRNAs exhibit pronounced specificity, with select miRNAs showing high enrichment or exclusive expression in muscle, collectively identified as myomiRs (Singh et al., 2020; Horak et al., 2016; McCarthy, 2011). This tissue-specific expression suggests that myomiRs play specialized roles in regulating the unique characteristics and functions of muscle cells, including their development, contraction, and metabolic properties (Kovanda et al., 2014). Several critical myomiRs—including miR-1, miR-133a, miR-133b, miR-206, miR-208a, miR-208b, miR-486, and miR-499—have been identified and extensively characterized (Singh et al., 2020). Other miRNAs, which are expressed both in muscle and non-muscle tissues, play vital roles in skeletal muscle biology by participating in a complex regulatory framework that orchestrates muscle development and functionality (Güller and Russell, 2010; Singh et al., 2020; Wang J. et al., 2018). Examples of such miRNAs include miR-23, miR-24, and miR-181 (Zhang and Chen, 2018). The existence of both specialized myomiRs and more broadly expressed miRNAs within skeletal muscle indicates a sophisticated regulatory system where both unique and general mechanisms contribute to the precise control of gene expression in this tissue (Singh et al., 2020; Nie et al., 2015; Soares, 2012).

Among the pivotal miRNAs in skeletal muscle development, miR-1 and miR-133 are co-transcribed from shared genomic loci and execute different, at times antagonistic, functions during the course of myogenesis (Ju et al., 2015; Mizbani, 2015). The promotion of myoblast differentiation by miR-1 occurs through its targeting of histone deacetylase 4 (HDAC4), a transcriptional repressor that inhibits the expression of muscle-specific genes. Additionally, miR-1 can inhibit the proliferation of cardiomyocytes by targeting the transcription factor Hand2 (Ju et al., 2015; Luo et al., 2019; Mitchelson and Qin, 2015; Zhang et al., 2015). miR-1 plays a role in a negative feedback loop regulating myocyte differentiation, achieved by its targeting of serum response factor (SRF) (Ju et al., 2015; Coletti et al., 2016). miR-1 influences metabolic flexibility within skeletal muscle beyond differentiation, particularly through the regulation of pyruvate metabolic pathways (Ismaeel et al., 2024). miR-1 has been identified to inhibit Telokin expression in cardiac muscle, where Telokin functions as a smooth muscle-restricted suppressor of myosin light chain 2 (MLC2) phosphorylation (Heidersbach et al., 2013). Satellite cell differentiation is facilitated by miR-1 via the reduction of their proliferation and the modulation of Pax7, a vital regulator of satellite cell self-renewal (Chen et al., 2010). miR-1 exhibits increased expression during satellite cell differentiation and diminished expression following muscle injury (Chen et al., 2010; Friedrichs et al., 2011). Acute endurance exercise induces a notable elevation in miR-1 levels (Safdar et al., 2009). The fact that miR-1 and miR-133, with their contrasting roles, are transcribed together suggests a mechanism for ensuring a balanced and precisely controlled progression through different stages of myogenesis (Ju et al., 2015; Koutsoulidou et al., 2011; Nguyen et al., 2023).

Contrary to miR-1, miR-133 chiefly promotes the proliferation of myoblasts by downregulating SRF, a vital regulator of muscle cell differentiation. Through this interaction, a negative feedback loop is established as SRF promotes miR-133a expression, leading to amplified repression of SRF (Ju et al., 2015; Yu et al., 2014; Papaefthymiou, 2016). miR-133, while co-transcribed alongside miR-1, paradoxically inhibits the differentiation process of myoblasts (Deng et al., 2011). Muscle fiber type specification is regulated in part by the miR-133 family, encompassing miR-133a and miR-133b (Zhang and Chen, 2018). By targeting insulin-like growth factor 1 receptor (IGF-1R), miR-133 potentially regulates muscle growth by modulating the IGF-1 signaling pathway (Huang et al., 2011). By targeting Prdm16, miR-133 is implicated in governing the brown adipose differentiation pathway of skeletal muscle satellite cells. It directly and negatively regulates NFATc4, a transcription factor involved in various cellular processes (Xie et al., 2016; Yin et al., 2013). In bronchial smooth muscles, miR-133a negatively regulates RhoA, a small GTPase involved in cell contraction. Furthermore, miR-133 inhibits Runx2, a transcription factor crucial for bone formation. Its expression is downregulated by nicotine, leading to the upregulation of TGF-β1 and TGF-βRII (Xie et al., 2016; Pechkovsky et al., 2010; Chen et al., 2015). miR-133 is abundantly expressed during muscle development and is part of bicistronic clusters with both miR-1 and miR-206 (Ju et al., 2015; Crocco et al., 2024). Acute endurance exercise induces an upregulation of miR-133a levels, mirroring the increase seen in miR-1 (Safdar et al., 2009; Nie et al., 2016). miR-133a and miR-133b, found within exosomes derived from muscle, likely contribute to communication mechanisms in the local skeletal muscle environment (Mytidou et al., 2021).

Another critical myomiR in skeletal muscle development is miR-206, which exhibits specific expression in skeletal muscle and plays a significant role in promoting myoblast differentiation (Ma et al., 2015). miR-206 achieves this by repressing the expression of connexin 43 (Cx43), a gap junction protein that reduces electrical coupling between muscle fibers, thereby facilitating terminal differentiation (Ju et al., 2015; Azzimato, 2014; Li et al., 2017). The targeting of critical genes like DNA polymerase α1 (Polα1), Pax7, follistatin-like 1 (Fstl1), and utrophin (Utrn) contributes to the inhibition of proliferation and the facilitation of differentiation in muscle cells. miR-206 is tightly regulated by MyoD and MyoG, transcription factors that are critical to the progression of myogenesis (Ju et al., 2015; Rosenberg et al., 2006; Megeney and Rudnicki, 1995; Kablar et al., 2003). miR-206 holds a key function in skeletal muscle regeneration subsequent to injury, extending beyond its developmental role. By promoting differentiation and fusion, it drives the maturation of satellite cells, the endogenous muscle stem cells, into myofibers (Liu et al., 2012). miR-206 promotes myogenesis by downregulating a set of inhibitory regulators, notably Pax7, Notch3, and Igfbp5 (Chen et al., 2010). miR-206 has been shown to play a protective role in Duchenne muscular dystrophy (DMD) by reducing the rate at which the disease progresses. The expression of miR-206 is elevated in satellite cells subsequent to muscle injury and continues to increase during the progression of Duchenne muscular dystrophy (Ma et al., 2015; Gr et al., 2009; Bulaklak, 2017). miR-206 is involved in the innervation of myofibers by regulating the synthesis of Cx43 (Mytidou et al., 2021). Parallel to miR-1, miR-206 aids satellite cell differentiation by restricting their proliferation and targeting Pax7, with upregulated expression in differentiation phases and downregulation following muscle injury (Chen et al., 2010; Aráne et al., 2021). Interestingly, miR-206 can exhibit a dual role in regulating utrophin A expression, oscillating between direct repression and activation depending on the cellular context (Amirouche et al., 2014). It exerts its effects by modulating multiple mRNAs and proteins that contribute to favorable adaptations within dystrophic muscle tissue (Amirouche et al., 2017). The multifaceted role of miR-206 underscores its importance in maintaining muscle tissue throughout the lifespan, contributing to both development and repair processes.

miR-486 is another significant muscle-enriched miRNA that participates in myogenesis signaling networks (Zhang and Chen, 2018). Its expression, modulated by important myogenic transcription factors like MRTF-A, SRF, and MyoD, highlights its integration within the overarching transcriptional mechanisms of muscle development. miR-486 facilitates the activation of the phosphoinositide-3-kinase (PI3K)/Akt pathway, fundamental for muscle growth and homeostasis, by targeting and downregulating its suppressors, phosphatase and tensin homolog (PTEN) and Foxo1a (Small et al., 2010; Qin et al., 2013; Qiu et al., 2024; Xu et al., 2012). The inhibition of PTEN by miR-486 is a key mechanism underlying its vital contribution to cardiomyocyte survival. In muscular dystrophy, lowered miR-486 expression is evident, but its transgenic elevation in animal models exhibits the potential to reverse aspects of the dystrophic phenotype. Furthermore, miR-486 plays a role in regulating systemic inflammation by influencing the levels of circulating cytokines and chemokines (Wang R. et al., 2022; Sun et al., 2019; Zhu et al., 2019). Interestingly, circulating levels of miR-486 are downregulated in response to exercise (Aoi et al., 2013). Regulated by estradiol, miR-486 may be a contributing factor to the observed sex-based distinctions in cancer-associated muscle pathologies. miR-486 supports myotube development during myoblast differentiation by inhibiting myocardin-related transcription factor A (MRTF-A) expression (Wang et al., 2021; Olivieri et al., 2014; Nielsen et al., 2014). miR-486 is vital for sustaining normal muscular function and limits the expression of transcripts linked to dystrophic pathophysiology (Samani et al., 2022). The regulation of miR-486 by multiple key transcription factors underscores its central role in muscle growth and adaptation, potentially linking mechanical stimuli and developmental signals to downstream effects on muscle mass and function.

**FIGURE 3:**
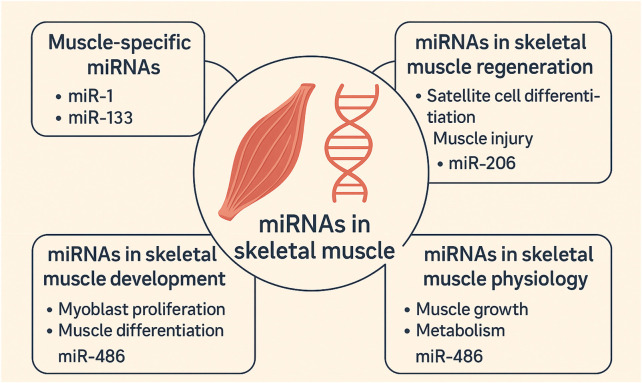
Scientific illustration depicting the major roles of miRNAs in skeletal muscle, including their involvement in tissue-specific expression, development, regeneration, and physiological regulation.

Other miRNAs, including miR-221 and miR-222, are implicated in skeletal muscle formation, with their suppressed expression during myoblast-to-myocyte transition highlighting their involvement in stage-specific progression (Cardinali et al., 2009). In skeletal muscle, aging is associated with decreased miR-451 expression, whereas its upregulation characterizes the differentiation of human myoblasts (Kirby et al., 2015; Munk et al., 2019; Figure 3; Table 2).

**TABLE 2:** Key MicroRNAs and exosomal miRNAs in Skeletal Muscle Development.

miRNA	Target Gene(s)	Function(s)	Reference
miR-1	HDAC4, SRF, Hand2, Telokin, Pax7	Promotes myoblast differentiation, regulates proliferation, involved in metabolic flexibility, represses Telokin in cardiac muscle, facilitates satellite cell differentiation	Ju et al. (2015)
miR-133	SRF, nPTB, IGF-1R, Prdm16, NFATc4, RhoA, Runx2	Promotes myoblast proliferation, inhibits differentiation, involved in muscle fiber type determination, influences IGF-1 signaling, involved in brown adipose determination	Ju et al. (2015)
miR-206	Cx43, Polα1, Pax7, Fstl1, Utrn, Notch3, Igfbp5	Promotes myoblast differentiation, suppresses proliferation, promotes muscle regeneration, slows DMD progression, involved in innervation	Ju et al. (2015)
miR-486	PTEN, Foxo1a, MRTF-A	Enhances PI3K/Akt signaling, essential for cardiomyocyte survival, reduced in muscular dystrophy, regulates inflammation, influences sex-specific muscle defects	Ju et al. (2015)
miR-221/222	—	Downregulated during myogenesis, role in myoblast to myocyte progression	Cardinali et al. (2009)
miR-451	—	Decreases with age, increases during myoblast differentiation	Kirby et al. (2015)

## Current limitations and future direction

Despite significant advances, several unresolved challenges hinder the translation of microRNA and exosomal microRNA research into clinical-grade therapeutics for skeletal muscle-related disorders.

One major obstacle lies in the isolation and purification of exosomes. Most protocols, including differential ultracentrifugation, yield heterogeneous vesicle populations that include microvesicles, apoptotic bodies, and protein aggregates. This complicates the attribution of function to exosome-specific miRNA cargo. While newer methods—such as size-exclusion chromatography, immunoaffinity-based capture, and microfluidic technologies—offer improved precision, there is currently no universally accepted standard, limiting reproducibility and inter-study comparison (Yang and Wu, 2018; Li et al., 2019).

Another critical challenge is the quantification and normalization of exosomal miRNAs. Techniques such as RT-qPCR, microarrays, and small RNA-sequencing are commonly used, but each introduces potential bias, sensitivity variation, and lacks reliable extracellular RNA reference controls. Distinguishing between truly exosome-encapsulated miRNAs and free-circulating or protein-bound miRNAs remains an experimental challenge requiring rigorous controls (Li et al., 2015; Moldovan et al., 2014; Siddika et al., 2020).

Validating the biological function of exosomal miRNAs in skeletal muscle is also technically demanding. Tracking vesicle uptake by target cells and demonstrating causal regulatory effects requires a combination of fluorescent labeling, loss- or gain-of-function experiments, and reporter assays—approaches that are rarely applied in concert (Boudna et al., 2024; Gupta et al., 2021). Even when uptake is shown, identifying which specific miRNA(s) mediate the observed effect remains a bottleneck, due to the multiplexed nature of exosomal cargo.

Moreover, delivering therapeutic miRNAs or antagomiRs in a stable, muscle-targeted, and immunogenically safe manner remains unresolved. While lipid nanoparticles, engineered exosomes, and viral vectors are under development, concerns over off-target effects, toxicity, immunogenicity, and regulatory hurdles remain (Messios et al., 2025; Kim et al., 2024; Gil-Cabrerizo et al., 2024).

From a translational perspective, a lack of human-relevant models poses another barrier. Most functional data are derived from rodents, which differ significantly from humans in muscle composition, metabolism, and miRNA expression patterns. *In vitro* studies often exclude the mechanical and paracrine complexity of the *in vivo* muscle niche.

Emerging technologies may help bridge these gaps. Single-vesicle profiling platforms (e.g., ExoView, nano-flow cytometry) are improving the resolution of cargo analysis. Advances in bioinformatics and multi-omics integration are enabling better mapping of miRNA-mRNA interactions and network regulation. Longitudinal clinical studies using miRNA panels as biomarkers are beginning to establish correlations with muscle health, aging, and therapeutic response.

Looking ahead, promising directions include.

• Development of synthetic or engineered miRNAs tailored to skeletal muscle disease targets
• Exploration of biomaterials (e.g., hydrogels, nanofibers) for localized delivery
• Investigation of miRNAs mediating muscle-organ crosstalk, particularly for metabolic disorders
• Sex- and age-specific miRNA profiling to uncover differential mechanisms and treatment opportunities

With continued interdisciplinary collaboration and technical innovation, miRNAs—especially in their exosomal form—hold significant potential for shaping the future of personalized and regenerative muscle medicine.

## Conclusion

This review emphasizes the evolving recognition of both intracellular and exosomal miRNAs as key regulators in skeletal muscle biology. From controlling fundamental signaling pathways to mediating intercellular communication, these small RNAs influence development, adaptation, and disease processes. Our perspective is that future progress will depend not only on refining molecular tools and delivery systems but also on deepening our systems-level understanding of miRNA networks in physiological and pathological contexts. As such, miRNAs—particularly in their exosomal form—represent both a scientific frontier and a translational opportunity in muscle research. In conclusion, intracellular and exosomal miRNAs represent a powerful regulatory layer in skeletal muscle physiology. Their roles in myogenesis, regeneration, metabolism, and disease response highlight their translational promise. However, realizing this potential requires resolving key experimental and therapeutic challenges. Continued integration of systems biology, emerging RNA technologies, and refined delivery platforms will be essential to fully unlock their value as biomarkers and clinical tools in skeletal muscle-related disorders.
